# Editorial: Molecular and cellular mechanisms of type 2 immunity-related disorders: allergic diseases and beyond

**DOI:** 10.3389/fimmu.2026.2014380

**Published:** 2026-10-20

**Authors:** Daniel P. Potaczek, Nathalie Acevedo

**Affiliations:** 1 Translational Inflammation Research Division & Core Facility for Single Cell Multiomics, Medical Faculty, Philipps-University of Marburg, Marburg, Germany; 2 Institute for Immunological Research, University of Cartagena, Cartagena, Colombia

**Keywords:** allergic airway inflammation (AAI), allergic conjunctivitis, allergic diseases, allergic rhinitis (AR), asthma, atopic dermatitis (AD), chronic rhinosinusitis (CRS), type 2 (T2) immunity/inflammation

Type 2 (T2) immunity is characterised primarily by the contribution of T2 cytokines, such as interleukins-4 (IL-4), -5 and -13, which play a key role in combating infections caused by parasitic worms and in the repair, remodelling and maintenance of tissue homeostasis (1, 2). In the case of regulatory disturbances or insufficient control, T2 immune responses contribute to the development of allergic diseases such as asthma, atopic dermatitis (AD), allergic rhinitis (AR), food allergy, allergic conjunctivitis and eosinophilic oesophagitis (3–5). The key cells involved in this process are type 2 T helper cells (Th2), group 2 innate lymphoid cells (ILC2s), mast cells (MCs), eosinophils, basophils and macrophages (6–8). However, the biology of the immune response is complex, and many aspects of it require further clarification (9–11).

This Research Topic (RT) included a collection of articles on the molecular and cellular mechanisms of T2 immunity-related disorders in human pathology. These articles varied in type, comprising both original research and comprehensive reviews. Furthermore, they addressed different aspects of the topic, including various clinical presentations.

The first group of articles focused on the mechanisms of asthma or other research issues relevant for asthma, with the first four works describing studies carried out on animal models of allergic airway inflammation (AAI) mimicking human asthma. Pacheco-Alba et al. investigated the effects of an ovalbumin (OVA)-based AAI model on the expression of the soluble and airway smooth muscle (ASM) isoforms of laminin, the most abundant components of the basement membrane, in guinea pigs. They found that soluble laminins in serum could serve as a marker to distinguish between OVA-induced AAI phenotypes. Whilst the α1 and α2 isoforms might be involved in ASM contraction, laminin β3 appeared to contribute to allergen-induced inflammation. Iriki et al. demonstrated that activation of the α7 nicotinic acetylcholine receptor, otherwise known for its anti-inflammatory effects, attenuated the development of AAI and suppressed the production of thymic stromal lymphopoietin (TSLP) by epithelial cells in a murine house dust mite (HDM)-based model. In turn, Mawatari et al. induced an OVA-based AAI model in wild-type (WT) mice and in animals in which Rab44, a large Rab GTPase involved in the membrane trafficking and differentiation of immune cells, was knocked out. They observed that Rab44 deficiency attenuated OVA-induced AAI by modulating immune responses and eosinophil function. In the fourth animal study, Schedel et al. investigated the molecular mechanisms governing the functional differentiation of steroid-insensitive T2 cytokine-producing CD8+ T cells (Tc2), a population of cells enriched in patients with severe asthma and correlating with disease severity. The comprehensive methodology included, among others, an OVA-induced AAI model with adoptive transfer of CD8+ T cells into CD8-deficient mice and comprehensive transcriptomic and epigenomic (histone 3 acetylation) analyses. They found that a central mechanism regulating the functional differentiation of Tc2 cells was based on a coordinated epigenetic and transcriptional remodelling.

The next paper reported the data obtained in a human study. Jakiela et al. aimed to shed some light on the mechanisms behind the beneficial clinical effects of bronchial thermoplasty (BT) in severe asthma. Although BT did not seem to profoundly change the immunological profile of the treated airways, it was found to be associated with a decrease in ASM thickness accompanied by only a modest increase in the expression of a subset of profibrotic genes and increased eosinophilia in bronchoalveolar lavage fluid. Within the framework of developing new approach methodologies for safety assessments that are more mechanism-focused and human-relevant, Hopkins et al. investigated epithelial responses to nebulised protease exposure, using a human air-liquid interface bronchial epithelial model. Their findings did indeed demonstrate the suitability of nebulised administration for assessing the effects of airborne proteases in bronchial ALI cultures. Furthermore, they also highlighted that profiling of microRNAs (miRNAs) derived from extracellular vesicles (EVs) could provide a powerful, non-invasive readout capturing early molecular perturbations not detectable by conventional functional assays alone. In addition, in a review article, Altynova et al. set out their views on the genetic and environmental risk factors for asthma. The environmental effects were also investigated in the study conducted by Bu et al. They carried out a comparative analysis of gut microbial immunoglobulin A (IgA) coating between an urbanised cohort of infants with a high prevalence of allergies and infants from a very traditional rural cohort with a very low prevalence of allergic disorders. Given the differences in the IgA coating of gut microbiota and the fact that human milk from communities with low rates of allergies exhibited higher IgA responses to infant commensals, including *Bifidobacterium infantis*, they concluded that differences in the IgA coating of gut microbiota might play a role in the development of allergic diseases in infancy.

The next several articles included in this RT dealt with AR or chronic rhinosinusitis (CRS). Liu et al. reviewed and discussed the facts on the immune sentinel function of nasal mucosal epithelial cells detecting allergens and initiating immune responses, thereby playing a pivotal role in the progression of AR. Bao et al. investigated in a murine model whether atmospheric particulate matter (PM) pollution, particularly fine PM (PM2.5), exacerbated HDM-induced AR and damage to the nasal epithelial barrier via the stimulator of interferon genes (STING) signalling pathway, which is thought to play an important role in chronic airway diseases. They found that PM2.5 disrupted the nasal epithelial barrier and, in synergy with HDM, exacerbated allergic inflammation by activating the STING/nuclear factor kappa-light-chain-enhancer of activated B cells (NF-κB) signalling pathway. Thus, STING was identified as a potential therapeutic target for environmentally induced allergic diseases. Carucci et al. investigated the links between nasal septum deviation (NSD) and AR in children in a multicentre, prospective study. They observed that AR might be more prevalent in children. Furthermore, they found that NSD could have a negative impact on the course of AR and, therefore, suggested that early detection and treatment of NSD could improve AR outcomes and reduce the overall disease burden. Milanković et al. investigated the subset composition of γδ T cells and their association with inflammatory remodelling in patients with eosinophilic CRS with nasal polyps (eCRSwNP). They found that progressive remodelling of γδ T cells in the sinonasal mucosa in eCRSwNP was associated with T2 inflammatory activity, tissue eosinophilia and the severity of the disease. Sumaily et al. carried out a systematic review and a meta-analysis to investigate the role of vitamin D in the prevention and treatment of CRS. They found that vitamin D deficiency was strongly associated with a more severe course of the disease and an increased risk of recurrence in CRS. Furthermore, vitamin D supplementation appeared to offer clinical benefits and could serve as a promising add-on to standard treatment for CRS.

The next two articles examined the links between AR and other conditions. Pu et al. conducted a comparative study of nasopharyngeal swabs-based transcriptomics in children with adenoid hypertrophy (AH) with or without concomitant AR, two conditions frequently occurring together in this age group. Interestingly, they found that AH was characterised primarily by a dysfunction of the epithelial barrier and an activation of the innate immune system associated with interleukin 36 (IL-36), whilst the accompanying AR was additionally associated with enhanced ciliogenesis and epithelial remodelling linked to T2 inflammation. Although AD and AR had been known to commonly coexist in childhood and to result in a significant clinical burden, previously available mechanistic models had not been able to sufficiently clarify this phenomenon. Thus, Mu et al. proposed the systemic epithelial-immune-neural framework as an operational and falsifiable model for investigating paediatric AD-AR comorbidity. In their conceptual article, they suggested a framework integrating four interacting mechanistic domains, such as the epithelial barrier and alarmin signalling, T2 immune activation, neuroimmune signalling, and microbiome regulation. With this, they established a structured basis for investigating the presence, potential mechanisms, heterogeneity and clinical relevance of cross-organ coupling in the comorbidity of AD and AR in paediatrics. On this occasion, the next article, written by Saleh et al., also dealt with skin allergy. Taking into account that dermal ILC2s (dILC2s) are frequently studied in recombination activation gene 1 (*Rag1*)-deficient mice, in which the dILC2 compartment may itself be altered, they compared dILC2s from WT and *Rag1*-deficient mice by transcriptomic profiling, flow cytometric phenotyping, and functional analysis in a contact hypersensitivity model with adoptive lymphocyte transfer. They found that a *Rag1* deficiency indeed permanently altered dILC2s in the skin and influenced contact hypersensitivity; this should be taken into account when interpreting models of skin inflammation in immunodeficient mice.

Returning to respiratory disorders, the next two papers were literature reviews covering fairly broad topics. The article by Zhu et al. described the mechanistic principles underlying the IL-33–ILC2 axis and its role in bronchopulmonary dysplasia, asthma, chronic obstructive pulmonary disease, pulmonary fibrosis and viral respiratory tract infections. In turn, Szatkowski et al. comprehensively presented and discussed biologic therapies targeting T2 inflammation in nonsteroidal anti-inflammatory drug-exacerbated respiratory disease, an inflammatory airway disease characterized by adult-onset asthma, CRSwNP and hypersensitivity reactions to cyclooxygenase-1 inhibitors.

The other three articles in our RT focused on the subject from the perspective of allergens. Urrego et al. aimed to produce, purify and characterise the physicochemical properties of recombinant isoforms of the *Blomia* (*B.*) *tropicalis* HDM allergen Blo t 2 (2.2. and 2.5), as well as to assess their clinical relevance and association with asthma phenotypes in Latin American populations, in which *B. tropicalis* is a major source of inhalant allergens. A detailed physicochemical and immunological characterisation proved the utility of highly stable, easily purifiable and soluble recombinant Blo t 2 isoforms. In addition, rBlo t 2 exhibited varying sensitisation patterns across populations in Brazil, Colombia and Ecuador and demonstrated its potential as a sensitisation biomarker for severe asthma in Brazilian adults, supporting its inclusion in future molecular diagnostic panels. Given the limited knowledge regarding the epigenetic effects of *B. tropicalis*, one of the main triggers of allergic asthma, Llinás-Caballero et al. investigated, in turn, the effects of the *B. tropicalis*-induced AAI model on DNA methylation in mouse lung tissue. They found that exposure to *B. tropicalis* allergens was associated with changes in DNA methylation in the lungs in genes linked to cell adhesion and neuroinflammation pointing to key genes of neuroimmune cross-talk alarm. This in turn might confirm the hypothesis that exposure to *B. tropicalis* could disrupt the airway epithelial barrier and sensitise the local neuroendocrine network of the lungs, thereby significantly predisposing the tissue to hyperreactivity and allergic inflammation. Last but not least, Chi et al. studied nasal mucosal transcriptomics and serum metabolomics in a mouse model of AR induced with mugwort (*Artemisia vulgaris*), a predominant aeroallergen associated with AR in northern China. In the model group, transcriptomic analysis revealed a clear T2 immune response, serum metabolomics showed increased levels of 1-methylhistamine and an enrichment of arachidonic acid metabolism, whilst integrated analysis of the two omics datasets further pointed to arginine-related metabolism as a shared altered pathway. In other words, the results indicated that the mugwort-induced AR was accompanied by coordinated local and systemic changes, including activation of the immune system, disturbances in epithelial and ciliary function, and metabolic changes in serum.

We firmly believe that the articles in this RT were of interest to many scientists and medical practitioners working in the field of asthma and/or other allergies. The RT series continues under the title *“Molecular and cellular mechanisms of T2 immunity-related disorders: Allergic diseases and beyond: Volume II”*
